# ﻿Chromosomes of four species of dobsonflies, in the genus *Protohermes* (Megaloptera, Corydalidae, Corydalinae) from East Asia

**DOI:** 10.3897/compcytogen.19.146501

**Published:** 2025-04-16

**Authors:** Yoshinori Takeuchi, Koji Iizuka, Hiroyuki Koishi, Hidehiro Hoshiba

**Affiliations:** 1 Bohkai Junior High School, 1-1-33 Nishi-Akashi, Akashi, Hyogo, 673-0041 Japan Bohkai Junior High School Hyogo Japan; 2 Matsue 4th Junior High School, 1-16-1 Nishi-Ichinoe, Edogawa-ku, Tokyo, 132-0023 Japan Matsue 4th Junior High School Tokyo Japan; 3 College of Agriculture, Tamagawa Univesity, 1-1 Tamagawagakuen, Machida, Tokyo, 194-8610 Japan Tamagawa Univesity Tokyo Japan

**Keywords:** Chromosomes, dobsonflies, *Protohermes*, sex chromosomes, XX/Xy_p_

## Abstract

We analyzed chromosomes of four species of East Asian dobsonflies (Megaloptera: Corydalidae): *Protohermesgrandis* (Thunberg, 1781), *P.immaculatus* Kuwayama, 1964, *P.disjunctus* Liu, Hayashi et Yang, 2007 and *P.costalis* (Walker, 1853). The chromosome number in all species was 2n = 24, consisting of 11 pairs of autosomes plus the XX chromosomes in females and the Xy_p_ in males. The karyotype of *P.immaculatus* which occurs in near the central part of the Ryukyu Islands, and is a vicarious species of *P.grandis*, was similar to the karyotype of *P.grandis*. On the other hand, the karyotype of *P.disjunctus*, which is from the Sakishima Islands, and is a vicarious species of *P.costalis*, resembled that of *P.costalis*.

The X chromosomes are submetacentric, while the Y is the smallest, dot-like chromosome of the set. The sex chromosomes of the first meiotic metaphase (MI) spermatocytes in all species invariably appear as a bivalent-like structure known as parachut bivalents Xy_p_, suggesting that the species in this genus share a common sex-bivalent mechanism.

## ﻿Introduction

Megaloptera is composed of two families, Corydalidae and Sialidae (= alderflies). Corydalidae is further divided into two subfamilies, Corydalinae (= dobsonflies) and Chauliodinae (= fishflies). Corydalinae larvae are aquatic, inhabiting deeper waters in streams and rivers and using tracheal gills on the ventral surface of their abdomens to absorb dissolved oxygen. On the other hand, Chauliodinae larvae live near the banks of rivers and are primarily air breathers, using a pair of respiratory tubes on the dorsal surface of the abdomen. Studies on Megaloptera chromosomes have been reported by Itoh (1933a, b), Hughes-Schrader (1980), and Takeuchi et al. (2002, 2012a, b). Two species of Corydalinae, one each from Japan and North America, both had chromosome number 2n = 24 (22+XY) (Hughes-Schrader 1980; Takeuchi et al. 2002). By contrast, four Japanese species of Chauliodinae had chromosome number 2n = 20 (18+XY) (Takeuchi et al. 2012a, b) and one species from North America had chromosome number 2n = 22 (20+XY) (Hughes-Schrader 1980). There are no reports on the chromosomes of Sialidae.

There are about 90 species of *Protohermes* Weele, 1907 recognized worldwide, with all occurring in Asia (Lacewing Digital Library 2025). Four of these species have been studied in this report: *Protohermesgrandis* (Thunberg, 1781), *P.immaculatus* Kuwayama, 1964, *P.disjunctus* Liu, Hayashi et Yang, 2007, and *P.costalis* (Walker, 1853). *P.costalis* is native to continental China, Taiwan, and India (Lacewing Digital Library 2025). The remaining three species are only known from Japan, with *P.disjunctus* known from the Yaeyama Islands, *P.grandis* from throughout Japan, and *P.immaculatus* from the Ryukyu Islands.

Specimens of *P.immaculatus* are small in size and considered a vicarious species of *P.grandis* (Hayashi 1989a). Vicarious species are considered to be closely related, having a common ancestor, but are geographically separated. Similarly, specimens of *P.disjunctus* are small in size and considered a vicarious species of *P.costalis* (Hayashi 1989b, c). Hayashi (1989a, b, c) reported that dwarfism resulted in dobsonflies from warm water temperatures and shortage of food on some islands. We studied chromosomes of these four *Protohermes* dobsonflies, using larval gonads. To date, of these four species the chromosome number has only been studied in *P.grandis* — 2n = 24 (Takeuchi et al. 2002). Our objective was to study and compare the karyotypes of these four *Protohermes* dobsonflies species and to discuss the finding in relationship to phylogenetic evolution caused by geographical isolation.

## ﻿Material and methods

### ﻿Insects

**Figure 1.**
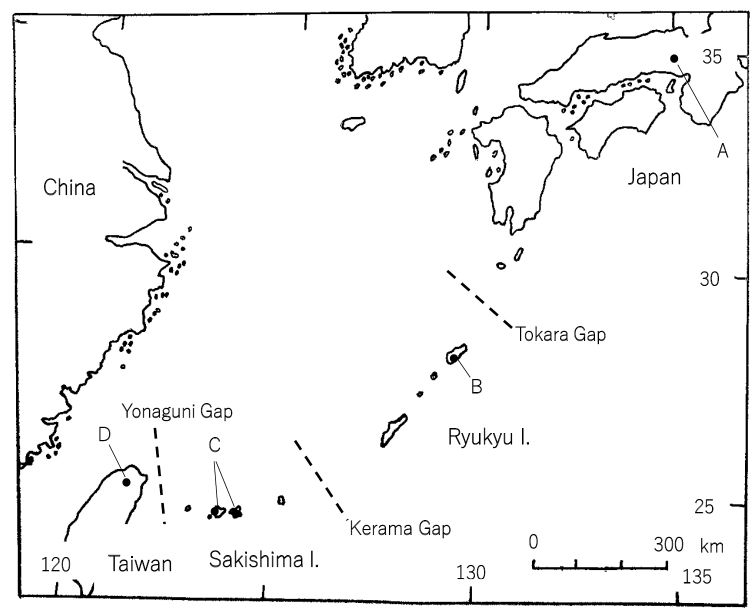
Locations where the larvae of four *Protohermes* were collected **A***Protohermesgrandis***B***P.immaculatus***C***P.disjunctus***D***P.costalis*. (See Table 1 for location details.)

Final-instar larvae of *Protohermesgrandis*, *P.immaculatus*, *P.disjunctus*, and *P.costalis*, were collected from June 1994 to February 1995 in rivers in Japan and Taiwan by the first author (Fig. 1). Collection sites, sampling dates, and the numbers of studied larvae are given in Table 1. In the field, larvae were placed individually in cups, filled with water and brought back to the laboratory alive. The larvae were fed aquatic insects until used in the experiment.

**Table 1.** Material used. Collection sites, sampling dates, and number of studied final-instar larvae of four *Protohermes* dobsonflies species.

*Protohermes* taxon	Sampling locality and date of collection	No. of studied larvae
*P.grandis*	Japan, Honshu, Hyogo Prefecture, Sugihara River 35°05'N, 134°53'E; VI.1994-VI.1995	13
*P.immaculatus*	Japan, Kagoshima Prefecture, Amami-Oshima Island, Kawauchi River; 28°17'N, 129°28'E; XI.1994	5
*P.disjunctus*	Japan, Okinawa Prefecture, Ishigaki Island, Nagura River; 24°24'N, 124°09'E; Iriomote Island, Takana River; 24°22'N, 123°54'E; VIII-XI.1994	6
*P.costalis*	Taiwan, Wulai, Tunhou Valley; 24°51'N, 121°29'E; II-1995	6

### ﻿Chromosome preparation

For all four species, the gonads of the final-instar larvae were used in the experiments. The dates of the experiment were from June 1994 to June 1995. Sex of each larva was determined by the width of the head (Takeuchi and Hoshiba 2012a). The method used in the present study is a modification of the Hoshiba et al. (1989) method. The tissues (testes and ovaries) were dissected from the larvae in 1% sodium citrate, and then placed in a colchicine solution (0.005%) for 30 minutes. The tissues were then placed on individual clean glass slides, after which three drops of a fixative I (F1: 3 parts glacial acetic acid, 3 parts of 99% ethanol, 4 parts of water) were added using a pipette. Next, a tiny drop of dissociation solution (1 part of 30% lactic acid diluted in glacial acetic acid and 2 or 3 parts of F1) was put on the tissue. The tissue samples were dissociated using a needle for 10–15 seconds. Then, two drops of another second fixative (F2: 1 part of glacial acetic acid: 1 part of 99% ethanol) were added to the cells by pipette, after which excess fixative and dissociation solution were removed with filter paper. Each slide was then dried, but just before the slide was completely dry, a drop of glacial acetic acid was added to the cells. To complete drying, the slides were then placed in an incubator at 40–50 °C overnight.

### ﻿Chromosome staining

The chromosomes were stained with 3% Gimsa’s solution in Sorensen’s phosphate buffer at pH 6.8 for 20 min (Hoshiba et al. 1989).

### ﻿Microscopy and imaging

Microscopic photography of chromosomal preparations was performed using an optical microscope (OL-IM) connected to a Microflex Afx-dx (both manufactured by Japan Optical Industry Co., Ltd.). Photographs of selected chromosome spreads were made using a 100× oil immersion lens. Photographs were taken using Mini-copy film ISO25 and Sencia ISO100 (both manufactured by Fujifilm Co., Ltd.) and printed on Fuji WP FM2~3 photographic paper.

## ﻿Results and discussion

**Figure 2.**
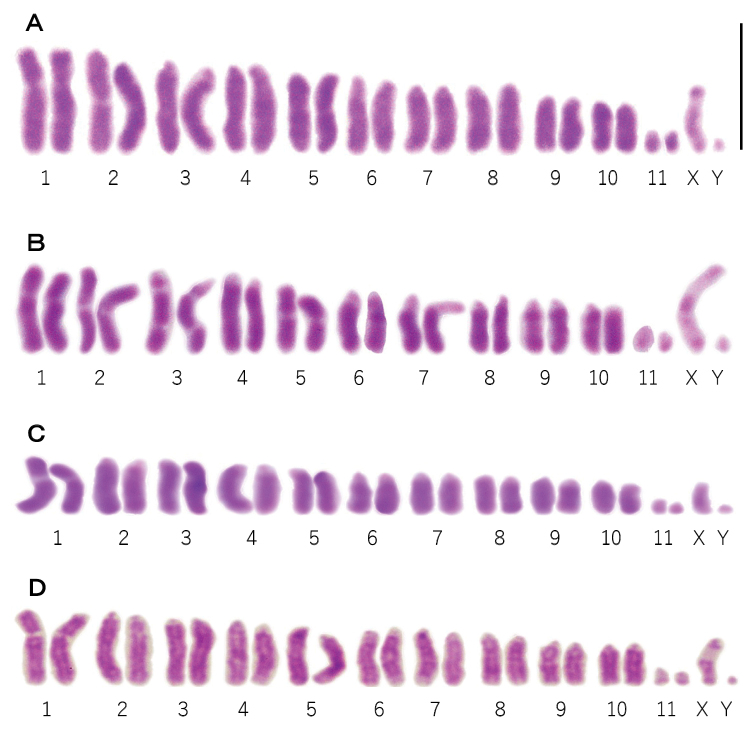
Karyotypes from spermatogonia of four species of dobsonflies in the genus *Protohermes***A***P.grandis* (male: 2n = 22 + XY) **B***P.immaculatus* (male: 2n = 22+XY) **C***P.disjunctus* (male: 2n = 22 + XY) **D***P.costalis* (male: 2n = 22 + XY). Scale bar: 5 μm.

The karyotypes were described following the nomenclature of Levan et al. (1964). The number of chromosomes for all four species was 2n = 24 (11 autosomal pairs +XX in females and 11 autosomal pairs + XY in males, Fig. 2). Takeuchi et al. (2002) reported that the autosomes of *P.grandis* consisted of one pair of large submetacentric chromosomes (no. 1), two pairs of metacentric chromosomes (no. 2 and 5), seven pairs of telocentric chromosomes (no. 3, 4, 6, 7, 8, 9, and 10), one pair of small chromosomes (no. 11) and the sex chromosomes consisting of a small submetacentric X chromosome and a small dot-like Y chromosome (Fig. 2A). In the present study, the autosomes of *P.immaculatus* consisted of one pair of large submetacentric chromosomes (no. 1), two pairs of metacentric chromosomes (no. 2 and 5), two pairs of subtelocentric chromosomes (no. 3 and 7), five pairs of telocentric chromosomes (no. 4, 6, 8, 9, and 10), one pair of small chromosomes and the sex chromosomes consisting of submetacentric X chromosome and a small dot-like Y chromosome (Fig. 2B). The X chromosome of *P.immaculatus* was about 5 µm long, which was considerably longer than the X chromosomes in the other three species (Fig. 2). A secondary constriction was observed in long-arm of chromosome number 3 of *P.immaculatus*. Overall, the karyotype of *P.immaculatus* was similar to that of *P.grandis* based on chromosome number and the position of the centromere on the chromosomes (Table 2). The autosomes of *P.disjunctus* consisted of one pair of large submetacentric chromosomes (no. 1), nine pairs of telocentric chromosomes (no. 2, 3, 4, 5, 6, 7, 8, 9, and 10), one pair of small chromosomes and the sex chromosomes consisting of a small submetacentric X chromosome and a small dot-like Y chromosome (Fig. 2C). The autosomes of *P.costalis* consisted of one pair of large submetacentric chromosomes (no. 1), nine pairs of telocentric chromosomes (no. 2, 3, 4, 5, 6, 7, 8, 9, and 10), one pair of small chromosomes and the sex chromosomes consisting of a small submetacentric X chromosome and a small dot-like Y chromosome (Fig. 2D). Overall, the karyotype of *P.disjunctus* was similar to that of *P.costalis* based on chromosome number and the position of the centromere on the chromosomes (Table 2).

**Table 2.** Chromosome numbers of Corydalinae (Megaloptera) species so far studied with karyotype descriptions. LM: large metacentric; LSM: large submetacentric; M: metacentric; SM: submetacentric; ST: subtelocentric; T: telocentric; dot: a very small chromosome.

Species	Chromosome number (2n)	Morphology			Method	Authors
		Autosomes	X	Y		
CORYDALINAE (Dobsonflies)						
*Protohermesgrandis*	24	1LSM+2M+7T+1dot	SM	dot	drying-1*^1^	Takeuchi et al. 2002
					drying-2*^2^	Present study
*Protohermesimmaculatus*	24	1LSM+2M+2ST+5T+1dot	SM	dot	drying-2*^2^	Present study
*Protohermesdisjunctus*	24	1LSM+9T+1dot	SM	dot	drying-2*^2^	Present study
*Protohermescostalis*	24	1LSM+9T+1dot	SM	dot	drying-2*^2^	Present study
*Corydaluscornutus*	24	1LM+1M+8T+1dot	SM	dot	squash	Hughes-Schrader 1980

*^1^ = method by Kezer and Sessions (1979); *^2^ = method by Hoshiba et al. (1989).

According to the paleogeographic map in and around the Ryukyu Arc since the late Pliocene, non-marine water was represented in the inland region during 2–1.7 Mya. In addition, the Ryukyu Arc ran from Kyushu to Taiwan several times during 0.4–0.02 Mya (Kimura 1996). Since 0.02 Mya, the Ryukyu Islands have been formed by sea immersion and some gaps (Fig. 1). Although speciation occurred due to geographic isolation, karyotypes remained similar between *P.grandis* and *P.immaculatus*, as well as between *P.costalis* and *P.disjunctus*. The cytological data in the present study supports the current concepts on taxonomy and phylogeny that *P.immaculatus* is a vicariant in the Ryukyu islands of *P.grandis* and *P.disjunctus* is a vicariant in the Sakishima islands of *P.costalis*. In the dobsonflies there appears to be a link between phylogenetic evolution and karyotype similarity.

**Figure 3.**
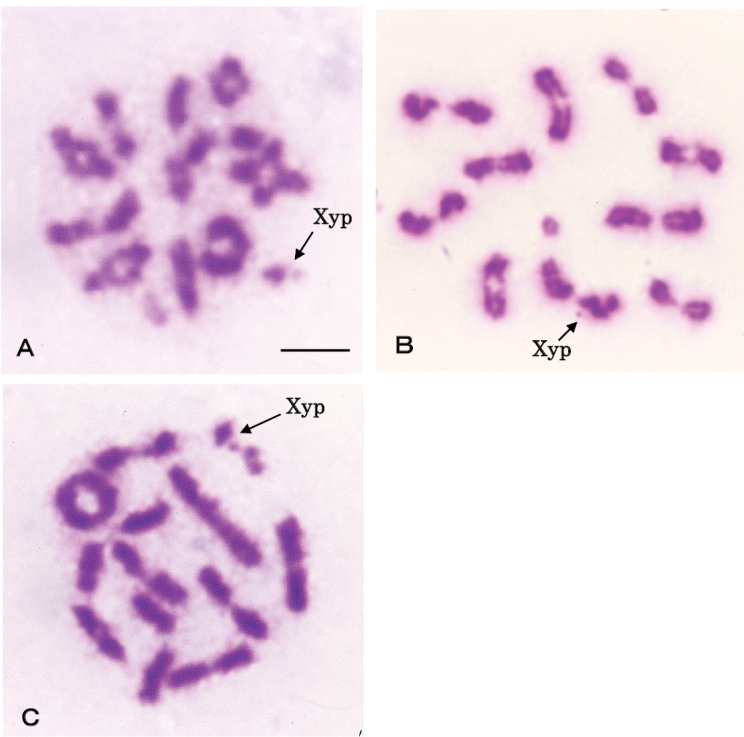
Male spermatocyte of three species of dobsonflies in the genus *Protohermes*. Arrows indicate X and Y chromosomes that form parachute-type bivalents (“Xy_p_”) **A***P.immaculatus***B***P.disjunctus***C***P.costalis*. Scale bar: 5 μm.

Takeuchi et al. (2002) reported the parachute-type bivalent “Xy_p_” sex chromosomes of *P.grandis*. We found similar Xy_p_ sex chromosomes in three additional *Protohermes* species in the present study (Fig. 3). This bivalent type of chromosomes has also been reported in two species of *Parachauliodes* fishflies (Takeuchi et al. 2012a), two species of *Neochauliodes* fishflies (Takeuchi et al. 2012b), and in the American fishfly species *Neohermesfillicornis* (Banks, 1903) (Hughes-Schrader 1980). This type of sex chromosome bivalent is also well-known and common in Coleoptera (Smith and Virkki 1978).

As in the present study, Hughes-Schrader (1980) reported that the North American dobsonfly *Corydaluscornutus* Linnaeus (1758) had 2n = 24 (11 autosomal pairs +XX in the female and 11 autosomal pairs + Xy_p_ in the male). This similarity between East Asian and North American Corydalinae demonstrates that chromosome number is highly conserved among dobsonflies on different continents.
